# Tractography-based DBS lead repositioning improves outcome in refractory OCD and depression

**DOI:** 10.3389/fnhum.2023.1339340

**Published:** 2024-02-07

**Authors:** Genevieve Basich-Pease, Natalya Slepneva, Adam C. Frank, Tenzin Norbu, Melanie A. Morrison, Leo P. Sugrue, Paul S. Larson, Philip A. Starr, A. Moses Lee

**Affiliations:** ^1^Weill Institute for Neurosciences, University of California, San Francisco, San Francisco, CA, United States; ^2^Department of Psychiatry and Behavioral Sciences, University of California, San Francisco, San Francisco, CA, United States; ^3^Department of Psychiatry and Behavioral Sciences, Keck School of Medicine of USC, Los Angeles, CA, United States; ^4^Department of Radiology and Biomedical Imaging, University of California, San Francisco, San Francisco, CA, United States; ^5^Department of Neurological Surgery, University of California, San Francisco, San Francisco, CA, United States; ^6^Department of Neurological Surgery, University of Arizona, Tucson, AZ, United States

**Keywords:** deep brain stimulation, OCD, anterior limb of the internal capsule, diffusion imaging, tractography, fMRI

## Abstract

Deep brain stimulation (DBS) of the anterior limb of the internal capsule (ALIC) has been used to treat refractory obsessive-compulsive disorder (OCD) and depression, but outcomes are variable, with some patients not responding to this form of invasive neuromodulation. A lack of benefit in some patients may be due to suboptimal positioning of DBS leads. Recently, studies have suggested that specific white matter tracts within the ALIC are associated with improved outcomes. Here, we present the case of a patient who initially had a modest improvement in OCD and depressive symptoms after receiving DBS within the ALIC. Subsequently, he underwent unilateral DBS lead repositioning informed by tractography targeting the ventrolateral and medial prefrontal cortex’s connection with the mediodorsal thalamus. In this patient, we also conducted post-implant and post-repositioning diffusion imaging and found that we could successfully perform tractography even with DBS leads in place. Following lead repositioning into tracts predictive of benefit, the patient reached responder criteria for his OCD, and his depression was remitted. This case illustrates that tractography can potentially be used in the evaluation and planning of lead repositioning to achieve therapeutic outcomes.

## Introduction

Deep brain stimulation (DBS) of the anterior limb of the internal capsule (ALIC) has been used to treat refractory obsessive-compulsive disorder (OCD) (Mallet et al., 2008; Denys et al., 2010; Luyten et al., 2016; Mosley et al., 2021) and depression (Malone et al., 2009; Dougherty et al., 2015; Bergfeld et al., 2016). However, approximately 30–40% of patients do not respond to DBS for OCD (Denys et al., 2020). Moreover, many of the initial studies using ALIC DBS to treat refractory depression have inconsistent outcomes (Malone et al., 2009; Dougherty et al., 2015; Bergfeld et al., 2016). It is possible that suboptimal positioning of DBS leads contributes to a lack of benefit in some patients. Recently, studies have suggested that positive DBS outcomes in OCD and depression are associated with modulation of white matter tracts coursing between the ventrolateral and medial prefrontal cortex (PFC), through the ALIC, and subcortically to the medial thalamus and subthalamic nuclei (STN) (Baldermann et al., 2019; Li et al., 2021).

Here, we report the case of a patient with DBS of the ALIC for OCD who underwent unilateral DBS lead repositioning after having an initial limited therapeutic response. Structural and diffusion-weighed imaging (DWI) were performed pre-operatively, post-implant, and following lead repositioning. We describe the patient’s history and structural neuroimaging to support the role of using tractography to optimize DBS surgical planning.

## Case description

The patient is a 53-year-old man with a history of severe, treatment-refractory OCD and co-morbid severe major depressive disorder (MDD). His harm-based OCD began in his teenage years, and he was formally diagnosed at age 28. His OCD primarily surrounds shame- and harm-based obsessions associated with extensive checking behaviors. At the time of evaluation, the patient described his OCD symptoms as near constant, feeling “intensely hopeless and anxious” to the point where he found it difficult to eat. He had not been going to work for 1–2 days each week due to the severity of his symptoms. In turn, the high level of distress from his OCD symptoms had caused him to feel depressed and isolated. He found it difficult to take pleasure in his daily activities, and his motivation to get out of bed and go to work was low.

Numerous medications, including multiple selective serotonin reuptake inhibitors, multiple serotonin and norepinephrine reuptake inhibitors, augmentation with multiple antipsychotics, clomipramine, and intravenous ketamine, were trialed to treat his OCD and MDD with limited benefit or intolerable side effects. He did not respond to repetitive transcranial magnetic stimulation for OCD or depression. He underwent extensive psychotherapy, including exposure-response prevention, with limited benefit. He also attended partial hospitalization programs and intensive outpatient programs, specializing in OCD with a limited response. Given his history of severe, treatment-refractory OCD and MDD, the patient was deemed an appropriate candidate for DBS targeting the ALIC for both disorders (Mallet et al., 2008; Malone et al., 2009; Denys et al., 2010; Dougherty et al., 2015; Bergfeld et al., 2016; Luyten et al., 2016; Denys et al., 2020; Mosley et al., 2021). Before DBS surgery, both his OCD and depression symptoms were in the severe to extreme range, with a Yale-Brown Obsessive Compulsive Scale (YBOCS) of 36 and a Patient Health Questionnaire-9 (PHQ-9) of 25.

**Figure 1:**
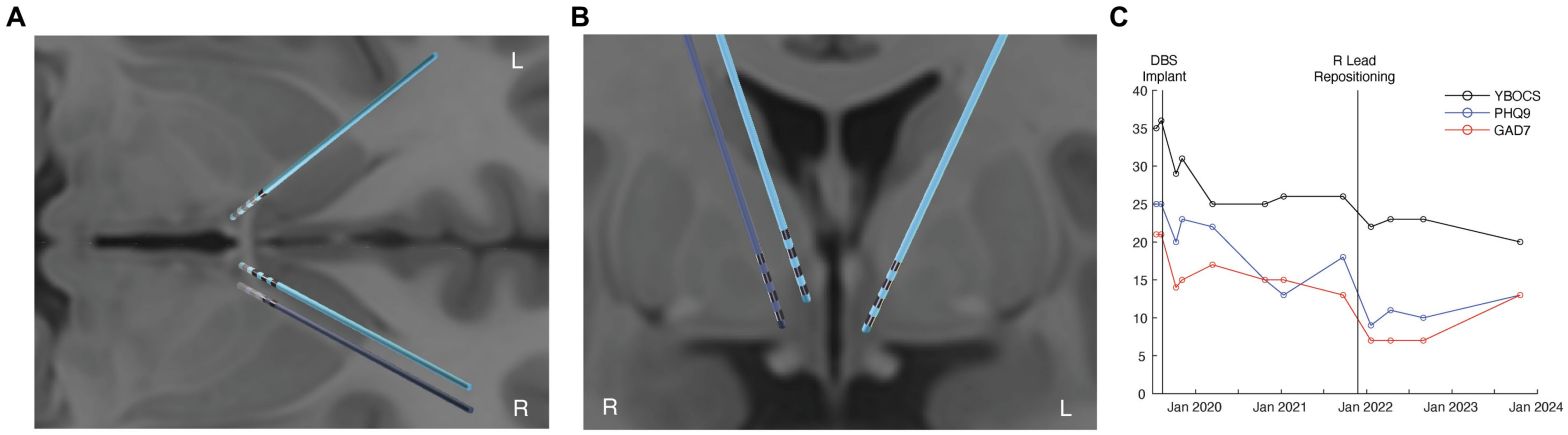
Improved OCD and depression outcomes from tractography-informed DBS repositioning. **(A)** axial view of the original (light blue) and repositioned DBS leads (dark blue). **(B)** coronal view of the original (light blue) and repositioned DBS leads (dark blue). **(C)** clinical improvement in OCD (YBOCS), depression (PHQ-9), and anxiety (GAD7) scores before DBS, after DBS, and following right lead repositioning.

Pre-operative T1-weighted structural magnetic resonance imaging (MRI) and DWI (55-direction HARDI, *b* = 2000) were acquired on a 3 T GE scanner with a eight-channel head coil for the purposes of surgical planning. Two quadripolar DBS leads (3,387; Medtronic, Minneapolis, MN) were stereotactically implanted within the bilateral ventral ALIC, with the middle contacts being targeted close to the bed nucleus of the stria terminalis (BNST). The right lead was positioned approximately 5 mm closer to the midline compared with the left lead position on the MRI (Figures 1A,B). The middle contacts of the right lead were positioned within the BNST. Standard stimulation programming was initiated 4 weeks after implantation. The patient experienced acute improvements in mood and anxiety with stimulation from the left lead in the C + 2- monopolar configuration at 7 mA. However, DBS using the right lead was discontinued after several months due to a lack of benefit even at high currents across multiple contacts. The patient achieved a partial benefit in OCD symptoms with a reduction in YBOCS to 25 (28% improvement compared to immediately before DBS) and improvement in depression (PHQ-9 reduced to 14; 40% improvement) after 1 year of treatment (Figure 1C). During this time, medications were not altered, and the patient was engaged in weekly ERP.

**Figure 2:**
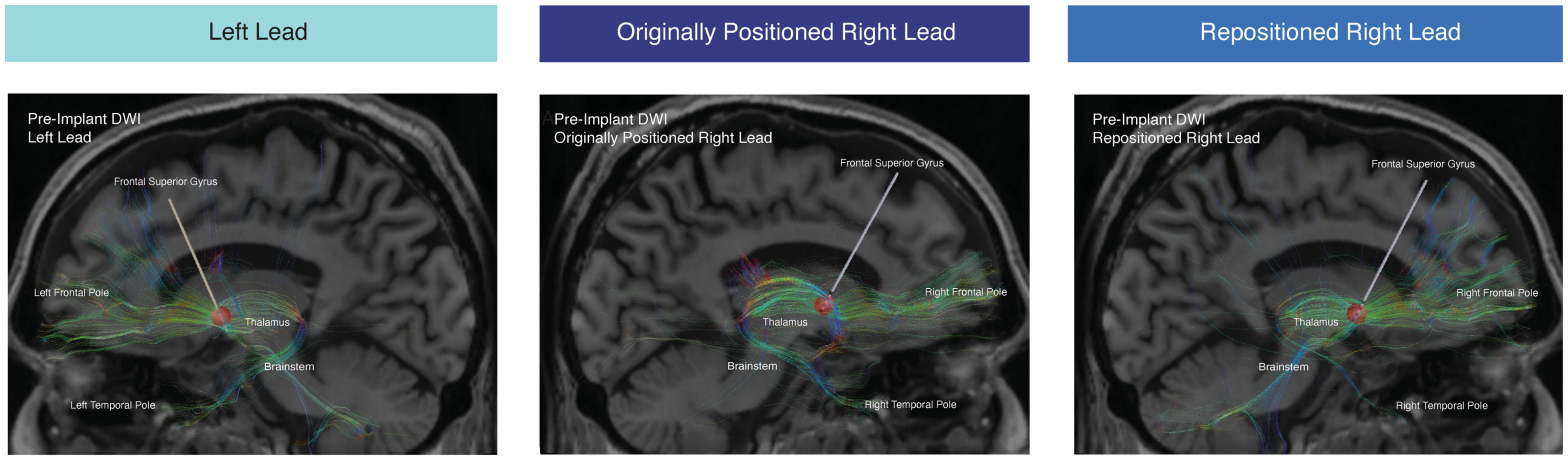
Tractography from estimated VAT. Tractography from the estimated VAT from the therapeutic active left contact (5 mA C + 2-) (left panel), right contact (5 mA C + 1-) from the originally positioned right lead (middle panel), and right contact from the repositioned right lead (right panel). Tractography was conducted with the pre-implant DWI.

There was a concern that the lack of benefit from the right lead was due to its medial location relative to the therapeutic left lead. Whole-brain tractography was performed from an estimated volume of activated tissue (VAT) from the left therapeutic C + 2- configuration using the Lead-DBS software package (Cieslak et al., 2021). The tractography was performed using the pre-implant DWI and post-operative T1-weighted structural MRI for lead localization. The left VAT demonstrated structural connectivity to the medial PFC, mediodorsal thalamus, and STN through tracts previously associated with benefit for OCD (Figure 2, left panel). However, the right VAT was medial to these tracts predictive of response. Instead, the right VAT was structurally connected with the stria terminalis and ansa peduncularis, tracts that project to the temporal lobe and amygdala (Figure 2, middle panel).

At approximately 2 years post-implantation, a right lead repositioning was attempted due to the patient’s significant residual OCD and mood symptoms. During the repositioning, the new lead was targeted along a trajectory that was informed by tractrography using the pre-implant DWI. Tractography was used to identify the anterior thalamic radiation (ATR) connecting the medial and ventrolateral prefrontal cortex with the mediodorsal thalamus. The right DBS lead was repositioned so the dorsal contacts were within the ATR based upon the tractography, mirroring the more lateral placement of the left therapeutic lead (Figures 1A,B).

Following repositioning and subsequent reprogramming, stimulation from the right C + 1- contact at 5 mA led to an acute improvement in mood and anxiety. The patient’s OCD improved by 39% (a reduction in YBOCS to 22) from before DBS. His depressive symptoms also improved by 64% (reduction in PHQ-9 to 9) relative to before DBS (Figure 1C). Additional improvement in OCD symptoms was observed at the last follow-up, 22 months after lead repositioning, with the last YBOCS being 20 (a reduction of 44% compared to prior to DBS implantation). While the patient did still have a moderate amount of residual symptoms, he noted that the benefit for his OCD from DBS was significant and meaningful to him, allowing him to work and engage in relationships, which was previously difficult given the nature of his obsessions. Importantly, his improvement in mood was also substantial; he noted that he was better able to cope in the face of stressors and had a sustained improvement in his motivation and energy with DBS.

**Figure 3:**
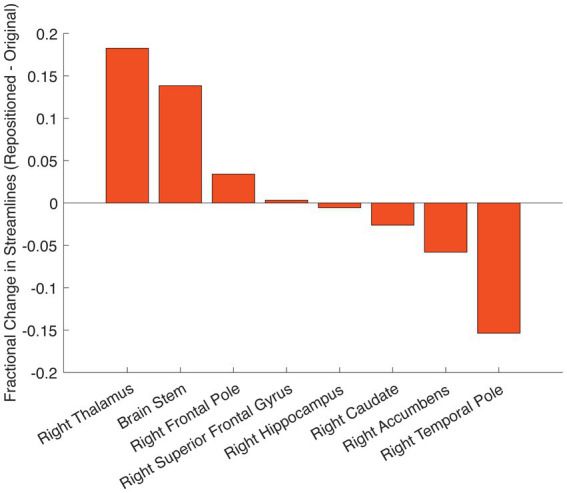
Change in structural connectivity following DBS repositioning. Change in streamline counts between the repositioned and originally positioned active contact on the right leads to different brain regions. Streamlines are derived from tractography using the pre-implant DWI.

Using the pre-implant DWI, we conducted tractography from the stimulation field corresponding with the repositioned right lead (Figure 2, right panel). After lead repositioning, there was an increase in the fraction of total streamlines from the right VAT to the right thalamus, brainstem, and right frontal pole. These tractography results are consistent with prior studies demonstrating that increased structural connectivity to these regions is correlated with improved DBS outcomes for OCD (Figures 2, 3) (Baldermann et al., 2019; Li et al., 2021). There was also decreased connectivity between the VAT and the right temporal lobe following repositioning, which has been associated with worse outcomes (Figures 2, 3).

**Figure 4:**
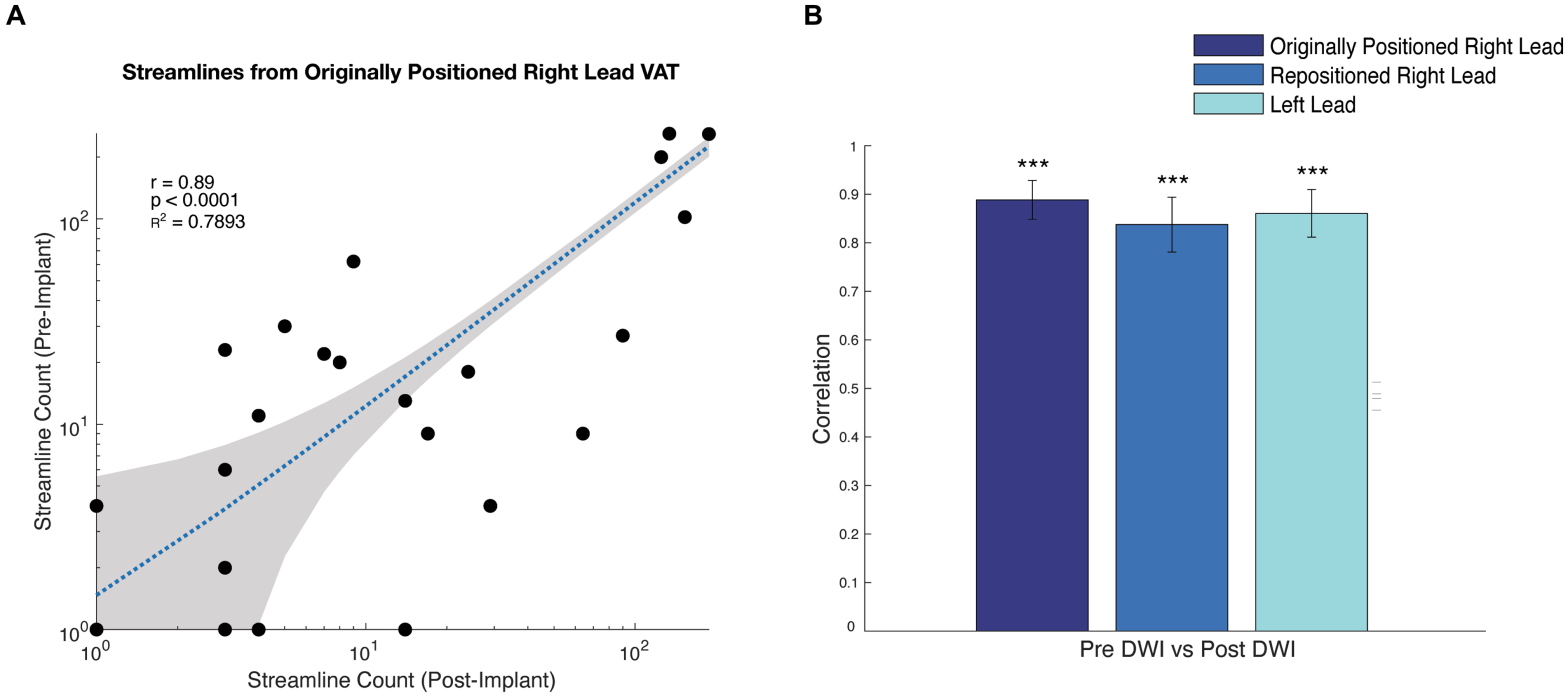
Correlation between tractography results from pre-implant, post-implant, and post-repositioning DWI. **(A)** Scatterplot of the streamline counts to each parcel for the original lead positioning using pre- and post-implant DWI. **(B)** bar graph showing the correlation coefficient of the streamline counts between parcellated regions and the VAT corresponding with the originally positioned right lead, repositioned right lead, and left lead comparing the pre-implant and post-implant DWI. *** *p* < 0.0001 based upon a *t*-test.

In this case, we were able to use personalized tractography to evaluate the need for DBS lead repositioning because DWI had been acquired prior to the DBS implant. However, in many cases, pre-implant DWI may not have been acquired. For this reason, we were interested in seeing if it was possible to perform reliable tractography using DWI that is acquired after DBS implantation with leads in place. With local IRB approval and written patient consent, we reacquired T1-weighted structural MRI and DWI (29-direction, b = 1,000) following the original DBS lead implantation as well as after the repositioning. We were able to successfully reconstruct tracts passing through the estimated VAT from the originally positioned right lead, the repositioned right lead, and the left lead, using both the post-implant and post-repositioning DWI with the DBS leads in place (Supp Fig S1). We validated the tractography using the post-implant DWI against the pre-implant results, demonstrating a strong correlation between the streamline counts from the VAT to other parcellated brain regions (Horn et al., 2019) (Figure 4, r_R Lead-Original_ = 0.89; *p* = 2.4 × 10^−39^, r_R Lead-Repositioned_ = 0.84; *p* = 6.7 × 10^−31^, r_L Lead_ = 0.86; *p* = 2.6 × 10^−34^). We also validated the tractography from the post-repositioning DWI against the pre-implant and post-implant tractography (Supplementary Figure S2). To the best of our knowledge, this is the first demonstration that tract reconstructions can be performed using post-implant DWI in a patient receiving DBS for OCD.

## Methods

### MRI

MRI scans were acquired on a 3 Tesla scanner (Discovery MR750, GE Healthcare, Chicago, IL). We collected T1- and T2-weighted structural scans and pre-implantation DWI (55-direction HARDI, *b* = 2000), T1-weighted structural and post-implantation DWI (29-direction, *b* = 1,000), and T1-weighted structural and DWI post-repositioning (29-direction, *b* = 1,000).

### Data analysis

DWI scans were preprocessed using QSIprep (Tournier et al., 2019). MP-PCA denoising as implemented in MRtrix3’s dwidenoise was applied with a five-voxel window, and then Gibbs unringing was performed using MRtrix3’s mrdegibbs (Andersson and Sotiropoulos, 2016). B1 field inhomogeneity was corrected using dwibiascorrect from MRtrix3 with the N4 algorithm. FSL’s eddy was used for head motion correction and eddy current correction (Friston et al., 2011). Shells were aligned post-eddy. Eddy’s outlier replacement was run. The DWI time series were resampled to ACPC, generating a preprocessed DWI run in ACPC space with 1 mm isotropic voxels. Using Lead-DBS (Cieslak et al., 2021), a MATLAB toolbox for DBS electrode reconstruction and simulation of DBS stimulation, T1, T2, and DWI scans were co-registered using SPM (Avants et al., 2008) and normalized using ANTs (Schoenecker et al., 2009; Yeh et al., 2010; Fonov et al., 2011), after which DBS electrodes were reconstructed and manually localized. White matter tracts were reconstructed from diffusion imaging data using generalized q-sampling (Baniasadi et al., 2020). The volume of activated tissue (VAT) was modeled for monopolar stimulation using FastField (Rushmore et al., 2022), and the VATs were used as seeds to generate connectivity to parcels from the Harvard-Oxford cortical and subcortical atlas (Horn et al., 2019).

## Discussion

While DBS is used to treat refractory OCD, approximately 30–40% of patients do not respond, and many still have significant residual symptoms after treatment has been optimized. Typically, the region targeted in DBS for OCD is the ventral ALIC, which encompasses a large area with associated structures including the nucleus accumbens, BNST, and overlying white matter tracts. Recently, studies have suggested that engagement of particular white matter tracts connecting ventrolateral and medial PFC through central ALIC to the thalamus and STN is an important predictor of benefit for outcomes for OCD and depression (Baldermann et al., 2019; Li et al., 2021; Gadot et al., 2023; Lai et al., 2023). Moreover, there is increasing interest in using personalized connectomes based on tractography to inform DBS targeting to account for individual differences in anatomy. Together, these findings suggest that one source of variability in DBS outcomes may be the positioning of leads relative to tracts predictive of improved outcomes.

Here, we report the case of a patient who achieved additional improvement after DBS lead repositioning targeted at white matter tracts predictive of DBS responsiveness. Before repositioning, many of the contacts were near the stria terminalis and ansa peduncularis projecting to the amygdala complex, and the patient had limited benefits for OCD and depression. Tractography was then performed using pre-implant DWI to identify white matter tracts connecting the medial PFC to the mediodorsal thalamus and STN, and DBS leads were repositioned to a region containing these tracts within the ALIC. Subsequently, the patient experienced additional improvement in his OCD and depressive symptoms, consistent with prior studies.

Furthermore, we demonstrated that it is possible to reproduce tract reconstructions with DBS leads in place. Given that MRI artifacts from leads can interfere with tract reconstruction, we validated that the structural connectivity estimates from stimulation VATs that we obtain with post-implant DWI are comparable to those estimated from a pre-implant scan. However, structural connectivity results derived from post-repositioning DWI had weaker correlations to pre-implant and post-implant DWI, specifically at the right repositioned lead, perhaps implying some change in white matter integrity following DBS repositioning surgery. The ability to reconstruct tracts using DWI with DBS leads in place is important given that pre-operative DWI is not always available in many cases.

Over a third of patients do not respond to DBS for OCD, highlighting the importance of determining how suboptimal lead placement or other factors contribute to poor outcomes. Here, we demonstrate that tractography can be used to evaluate whether leads are optimally positioned near white matter tracts predictive of benefit. Furthermore, we describe a case in which DBS lead repositioning informed by tractography improved clinical outcomes for a patient who was a partial responder. This case demonstrates the importance of anatomical targeting in clinical response. Future studies tracking lead repositioning outcomes in a larger cohort of OCD and DBS patients will be needed to validate these findings.

## Data availability statement

The raw data supporting the conclusions of this article will be made available by the authors, without undue reservation.

## Ethics statement

The studies involving humans were approved by the University of California, San Francisco. The studies were conducted in accordance with the local legislation and institutional requirements. The participants provided their written informed consent to participate in this study. Written informed consent was obtained from the individual(s) for the publication of any potentially identifiable images or data included in this article.

## Author contributions

GB-P: Formal analysis, Investigation, Methodology, Visualization, Writing – original draft, Writing – review & editing. NS: Methodology, Writing – review & editing. AF: Investigation, Writing – review & editing. TN: Investigation, Writing – review & editing. MM: Conceptualization, Funding acquisition, Methodology, Project administration, Resources, Supervision, Writing – review & editing. LS: Conceptualization, Funding acquisition, Investigation, Methodology, Writing – review & editing. PL: Investigation, Writing – review & editing. PS: Investigation, Methodology, Writing – review & editing. AL: Conceptualization, Data curation, Formal analysis, Funding acquisition, Investigation, Methodology, Project administration, Resources, Supervision, Visualization, Writing – original draft, Writing – review & editing.
